# Vascular endothelial mineralocorticoid receptors and epithelial sodium channels in metabolic syndrome and related cardiovascular disease

**DOI:** 10.1530/JME-23-0066

**Published:** 2023-09-13

**Authors:** Guanghong Jia, Michael A Hill, James R Sowers

**Affiliations:** 1 Department of Medicine-Endocrinology and Metabolism, University of Missouri School of Medicine, Columbia, Missouri, USA; 2 Dalton Cardiovascular Research Center, University of Missouri, Columbia, Missouri, USA; 3 Department of Medical Pharmacology and Physiology, University of Missouri School of Medicine, Columbia, Missouri, USA

**Keywords:** mineralocorticoid receptors, epithelial sodium channels, endothelial cells, metabolic syndrome

## Abstract

Metabolic syndrome is a group of risk factors that increase the risk of developing metabolic and cardiovascular disease (CVD) and include obesity, dyslipidemia, insulin resistance, atherosclerosis, hypertension, coronary artery disease, and heart failure. Recent research indicates that excessive production of aldosterone and associated activation of mineralocorticoid receptors (MR) impair insulin metabolic signaling, promote insulin resistance, and increase the risk of developing metabolic syndrome and CVD. Moreover, activation of specific epithelial sodium channels (ENaC) in endothelial cells (EnNaC), which are downstream targets of endothelial-specific MR (ECMR) signaling, are also believed to play a crucial role in the development of metabolic syndrome and CVD. These adverse effects of ECMR/EnNaC activation are mediated by increased oxidative stress, inflammation, and lipid metabolic disorders. It is worth noting that ECMR/EnNaC activation and the pathophysiology underlying metabolic syndrome and CVD appears to exhibit sexual dimorphism. Targeting ECMR/EnNaC signaling may have a beneficial effect in preventing insulin resistance, diabetes, metabolic syndrome, and related CVD. This review aims to examine our current understanding of the relationship between MR activation and increased metabolic syndrome and CVD, with particular emphasis placed on the role for endothelial-specific ECMR/EnNaC signaling in these pathological processes.

## Introduction

Metabolic syndrome refers to the presence of a cluster of risk factors specific for cardiovascular diseases (CVDs) (Gorini *et al.* 2018, Karthigan *et al.* 2022). According to the National Cholesterol Education Program (NCEP) Adult Treatment Panel III (ATP III) report, there are five major components of the metabolic syndrome which relate to the development of CVD: (i) abdominal obesity, (ii) insulin resistance (with or without the presence of glucose intolerance), (iii) Elevated triglycerides, (iv) Reduced high density lipoprotein cholesterol, and (v) Increased blood pressure. According to the NCEP ATP III guidelines, patients must meet three of the five criteria for metabolic syndrome to be diagnosed (Table 1) (Saklayen 2018). In 2005, the National Heart, Lung, and Blood Institute (NHLBI) and American Heart Association (AHA) conferred to establish a definition for the metabolic syndrome, which was also supported by the American Diabetes Association. Metabolic syndrome has become globally prevalent due to the parallel rise in the incidence of obesity, a situation that is expected to worsen in the coming decades (Ghaus *et al.* 2021). According to the World health organization, more than 19 billion adults were overweight, and over 650 million were obese in 2016 (Ghaus *et al.* 2021). Importantly, metabolic syndrome is not restricted to adults with approximately 1 million American adolescents (4% of the adolescent population) having been diagnosed as exhibiting metabolic syndrome according to the NCEP/ATP III diagnostic criteria (Saklayen 2018). The prevalence of metabolic syndrome and related CVD increases with age and is higher in women than men. In addition, ethnic and socioeconomic disparities have been noted with metabolic syndrome being more common among African Americans, Hispanics, and people with lower socioeconomic status (Saklayen 2018).

**Table 1:** NCEP ATP III proposed diagnostic criteria of metabolic syndrome.

Clinic measure	Diagnostic criterial (any three below)
Abdominal obesity	Men: >102 cm (40 in), women: >88 cm (35 in)
Fasting glucose	≥100 mg/dL
Triglycerides	≥150 mg/dL
High density lipoprotein	Men: <40 mg/dL, women: <50 mg/dL
Blood pressure	≥130 systolic or ≥85 mmHg diastolic
Other	None

NCEP ATP III, The National Cholesterol Education Program Adult Treatment Panel III.

Interactions between the established risk factors for metabolic syndrome, including hypertension, glucose intolerance, dyslipidemia, and obesity initiate and promote the pathophysiological changes associated with overt metabolic syndrome and CVD. Of relevance to this, inappropriate activation of the systemic and tissue renin–angiotensin–aldosterone systems often participate in these risk factors for developing metabolic syndrome. Furthermore, increased plasma aldosterone levels (Aroor *et al.* 2019, Vecchiola *et al.* 2020) and enhanced MR activation (DeMarco *et al.* 2015, Jia *et al.* 2016, Hulse *et al.* 2022) not only induce CVD but also contribute to glucose intolerance, dyslipidemia, and metabolic syndrome. Importantly, epithelial sodium (Na^+^) channels (ENaC), a downstream target of aldosterone/mineralocorticoid receptor (MR) signaling, are regarded as an important mediator in the pathogenesis of metabolic syndrome. Adding to the complexity of this pathway MRs are now appreciated to exist in various tissues outside the kidney, including adipose, skeletal muscle, and cardiovascular tissues. Related to this, recent studies have demonstrated that vascular-specific endothelial cell (EC) MR (ECMR)/endothelial ENaC (EnNaC) signaling plays an important in developing metabolic syndrome and CVD (Jia *et al.* 2016, Sowers *et al.* 2020). Here, we focus on reviewing recent experimental and clinical data related to ECMR/EnNaC to discuss their roles in metabolic syndrome and related CVD.

## Aldosterone, ECMR/EnNaC expression and activation

Aldosterone is principally synthesized in the zona glomerulosa of the adrenal cortex in response to renin–angiotensin system activation or high dietary potassium (Jia *et al.* 2021). Aldosterone is also produced in adipose tissue during obesity, or under conditions of overnutrition (Jia *et al.* 2021). Consistent with this, it has been shown that increased aldosterone synthase expression promotes both adrenal and fat aldosterone production in a calcineurin/nuclear factor of activated T cell-dependent manner (Rios *et al.* 2015). Inhibition of cholesteryl ester transfer protein induces increased aldosterone biosynthesis in a nicotinamide adenine dinucleotide phosphate (NADPH) oxidase (Nox)-dependent manner in adipocytes (Rios *et al.* 2015). One study has further demonstrated that human adipocytes are capable of aldosterone production in a partially angiotensin II dependent manner (Briones *et al.* 2012). In addition, adipose tissue-derived mineralocorticoid-releasing factors, including leptin and complement-C1q tumor necrosis factor-related protein 1, as well as angiotensin II, stimulate aldosterone release in human adrenocortical cells (Huby *et al.* 2015, Kawarazaki & Fujita 2016). Several studies further showed that the adipokine leptin has a direct role in promoting aldosterone synthase CYP11B2 expression and aldosterone release subsequently promoting cardiovascular dysfunction, leading to metabolic syndrome and related CVD (Huby *et al.* 2015, Kawarazaki & Fujita 2016). Thus, both adipose tissue and adrenal glands appear to be relevant sources of aldosterone in obesity. Indeed, our recent data have demonstrated that diet-induced obese mice exhibit significantly increased plasma aldosterone to levels of 3,166 pmol/L (Aroor *et al.* 2019) which can further activate MRs in vascular, skeletal muscle, and liver tissues, with resultant increases in systemic and tissue-specific insulin resistance (Jia *et al.* 2021).

**Figure 1:**
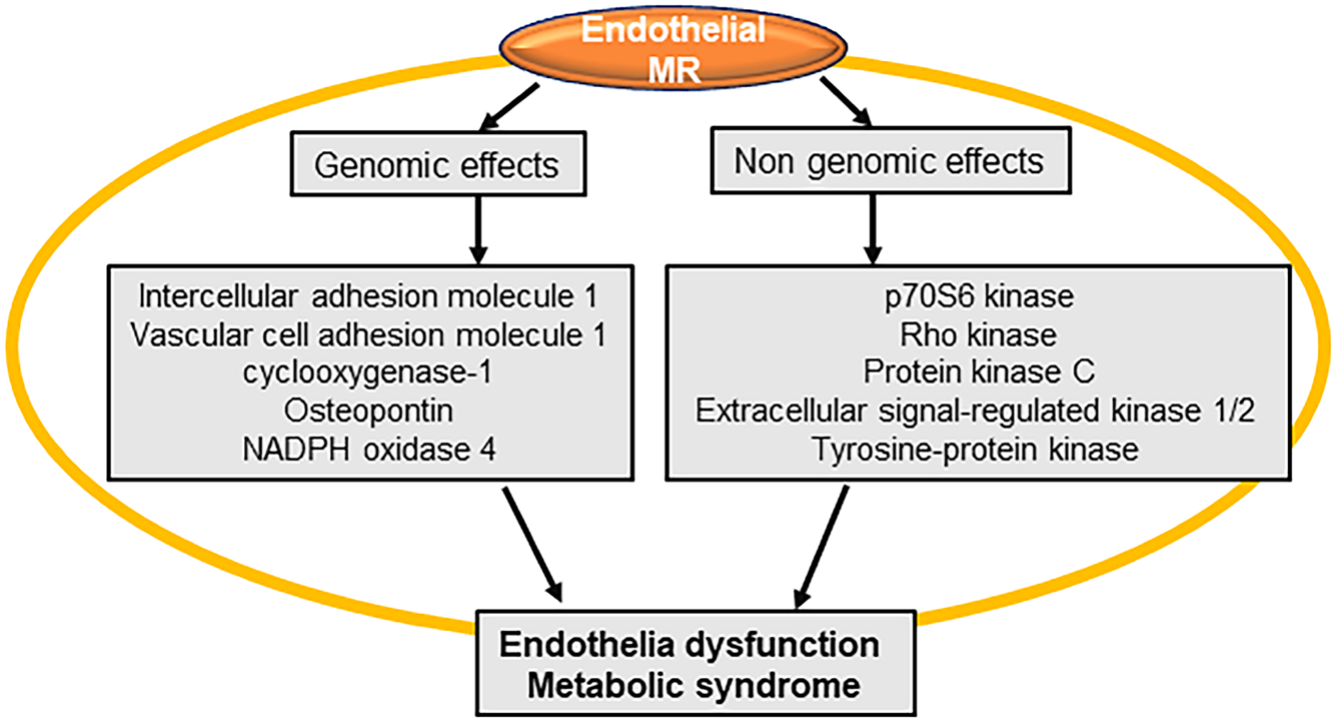
Genomic and nongenomic regulation of ECMR in endothelial dysfunction and metabolic syndrome.

Aldosterone activity is dependent on the binding and activation of the cytoplasmic/nuclear MR, which is an intracellular steroid hormone receptor and plays an important physiological role in regulating plasma volume, electrolyte homeostasis, and blood pressure (Jia *et al.* 2021). Recent data indicate that MRs are expressed and are functional in tissues outside of the kidney, including in pancreatic islets, adipose tissue, skeletal muscle, liver, immune cells, and cardiovascular tissue (Jia *et al.* 2021). For instance in ECs, upon being activated by aldosterone, the MR is translocated to the cell nucleus, where it regulates gene transcription and translation of proteins, such as intracellular adhesion molecule-1 (ICAM-1) and vascular cell adhesion molecule 1 (VCAM-1), by binding to DNA hormone/steroid stimulatory or negative response elements (Fig. 1) (Jia *et al.* 2021). Aldosterone also exerts rapid nongenomic effects through activation of kinases such as protein kinase C and Rho kinase, leading to mitochondrial dysfunction and excessive production of reactive oxygen species (ROS) in ECs (Fig. 1) (Jia *et al.* 2021). Meanwhile, MR activity is also regulated by cortisol. In tissues lacking 11β-hydroxysteroid dehydrogenase-2 such as adipose tissue, cortisol is typically the primary ligand for the MR since blood concentrations of cortisol are 100 to 1000 times higher than those of aldosterone levels (Jia *et al.* 2021). However, aldosterone is the primary ligand for MRs in both ECs and vascular smooth muscle cells because both vascular cells present 11β-hydroxysteroid dehydrogenase 2 that inactivates cortisol and allows the binding of aldosterone to the vascular MRs (Jaffe & Mendelsohn 2005, Caprio *et al.* 2008).

In the case of ENaC expression and regulation, aldosterone activates MRs that bind to the MR response element in the promoter regions of the genes encoding the ENaC subunits, leading to the activation of transcription of the ENaC genes and increased ENaC mRNA synthesis and protein expression (Jia *et al.* 2016, 2021). Meanwhile, aldosterone also activates MRs to promote activation of serum and glucocorticoid kinase 1 (SGK1), a key regulator in limiting ENaC ubiquitination and endocytosis through inhibition of E3 ubiquitin ligase (Nedd4-2), thus increasing the residence time of ENaC within the plasma membrane (Jia *et al.* 2018*a*,*b*, 2021). Related to this, increased Nedd4-2 activity promotes ENaC endocytosis and reduces ENaC content within the plasma membrane (Jia *et al.* 2018*a*,*b*, 2021). Our recent data further indicate that ECMR activates EnNaC expression and activation by binding to the ENaC promoter, which contains at least six of eight conserved nucleotides of the consensus sequence (NGNACAnnnTGTNCN) (Jia *et al.* 2016). Indeed, ENaC is a heterotrimeric ion channel consisting of α, β, γ,and δ subunits and has been extensively studied in respiratory and renal epithelial cells (Jia *et al.* 2018*a*,*b*). More recently, the existence of EnNaC has been demonstrated in the vascular ECs and contributes to vascular stiffness, hypertension, and coronary heart arterial disease (CAD) in metabolic syndrome (Jia *et al.* 2018*a*,*b*).

## ECMR and EnNaC in metabolic syndrome and related CVD

### Vascular ECMR/EnNaC in insulin resistance

**Figure 2:**
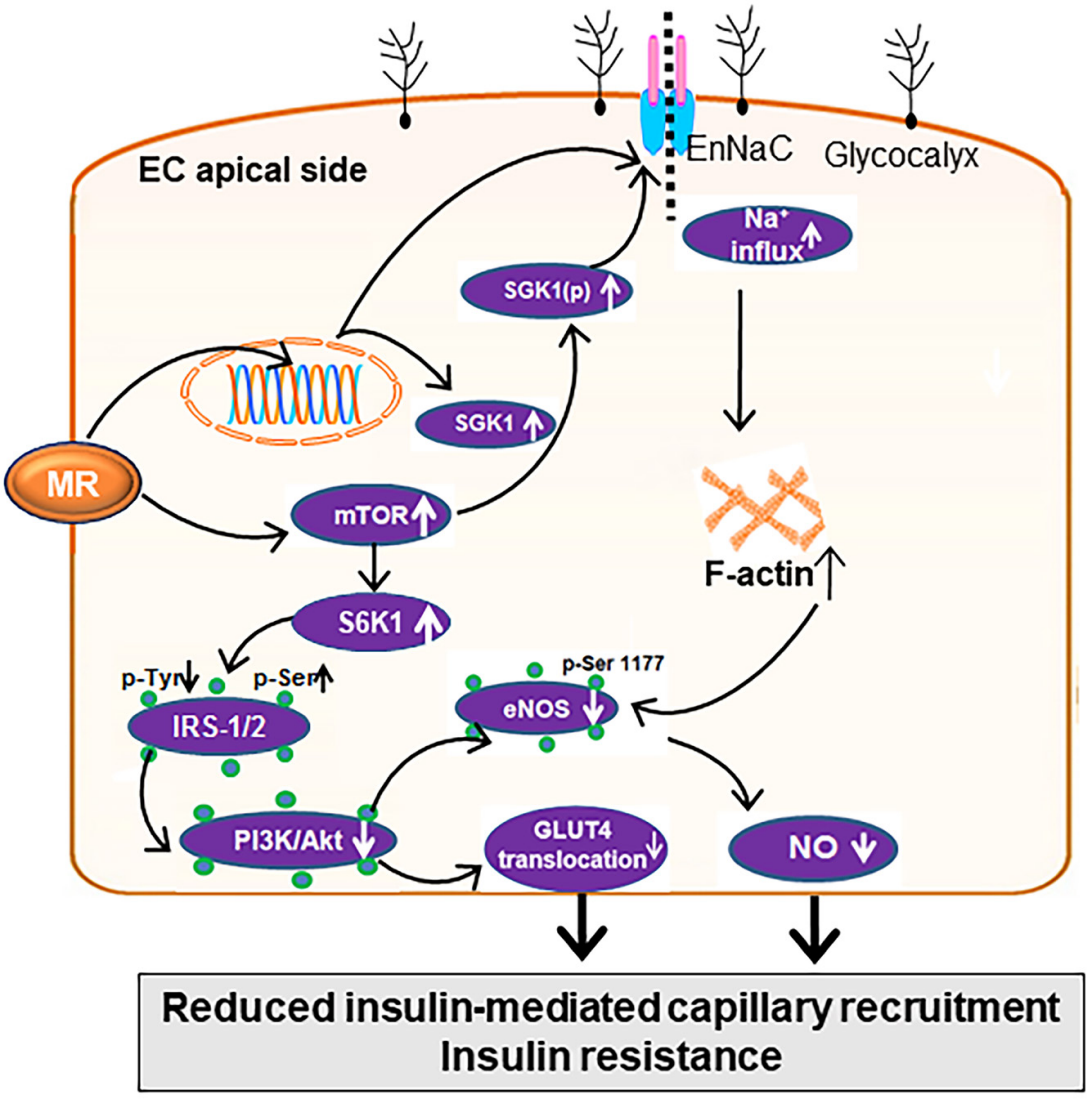
Enhanced ECMR signaling prompts EnNaC expression and activation, and associated reduced insulin-mediated capillary recruitment and insulin resistance.

Excessive activation of MR in ECs leads to vascular insulin resistance, which refers to the impaired responsiveness of blood vessels to the actions of insulin and related reduction in insulin-mediated capillary recruitment, glucose delivery, and subsequent tissue glucose uptake. In humans, vascular insulin resistance has been linked to various cardiovascular and metabolic disorders, including obesity, hypertension, atherosclerosis, and diabetes (Jia *et al.* 2021). In animal studies, aldosterone promotes vascular insulin resistance and dysfunction that were prevented with MR blockage (Wada *et al.* 2010, DeMarco *et al.* 2015). Inhibition of MRs or adrenalectomy improves insulin resistance in patients with primary aldosteronism (Catena *et al.* 2006) and MR antagonists have been shown to improve coronary endothelial function in diabetic patients (Garg *et al.* 2015). One of the key mechanisms underlying vascular insulin resistance is impaired signaling of the insulin receptor substrate 1 (IRS-1) pathway, which is a critical mediator of insulin signaling in the blood vessels. Impaired phosphorylation of IRS-1 and activation can lead to reduced nitric oxide (NO) production and impaired vasodilation (Jia *et al.* 2021). To this point, enhanced activation of ECMR may promote insulin resistance through activation of the mammalian target of the rapamycin (mTOR) signaling pathway (Jia *et al.* 2014). Chronic activation of mTOR/S6 kinase 1 (S6K1) by excessive nutrients, promotes insulin resistance in vascular through increased serine (Ser) phosphorylation (p) of the critical insulin signaling/docking molecule IRS-1, leading to impaired phosphoinositol 3 kinase (PI3-K) engagement and protein kinase B (Akt) stimulation, as well as reduced glucose transporter type 4 (GLUT4) translocation (Fig. 2). Stimulation of mTOR also induces activation of SGK1 and EnNaC expression/activation (Lang & Pearce 2016, Zhang *et al.* 2022). In this regard, ECs are normally protected by a well-developed glycocalyx that inhibits the access of Na^+^ into ECs and maintains vasodilation. Conversely, increased aldosterone levels activate ECMR to enhance SGK1 and inhibit EnNaC ubiquinization/degradation by upregulation of Nedd4-2, leading to increased EnNaC expression and plasma membrane abundance in ECs that leads to enhanced Na^+^ entry, polymerization of G-actin to F-actin, reduction of endothelium eNOS activity, NO production, and the development of EC stiffening and microvascular dysfunction (Fig. 2). Both ECMR and EnNaC activation lead to reduced endothelial NO synthase (eNOS) activation and NO bioavailability in association with reduced insulin-mediated capillary recruitment, glucose uptake, and systemic and tissue insulin resistance (Fig. 2).

### Vascular ECMR/EnNaC in arterial stiffness and hypertension

Vascular stiffening, characteri*z*ed by an abnormal increase in pulse wave velocity, is associated with metabolic syndrome. Linking this with the above discussion, obesity and insulin resistance lead to elevated aldosterone levels and MR activation which promotes arterial remodeling, and pathologically increased vascular stiffness, and predicts the risk of subsequent hypertension, CVD, and metabolic syndrome. Consistent with this an earlier clinical study demonstrated that patients with non-insulin-dependent diabetes or borderline abnormal glucose intolerance had stiffer arteries than those exhibiting normal glucose tolerance (Salomaa *et al.* 1995). The Framingham Offspring Study investigated the relationship between insulin resistance and the 4-year incidence of hypertension with this analysis indicating that in human subjects insulin resistance positively relates to the existence of hypertension (Arnlov *et al.* 2005). Moreover, inhibition of MRs with spironolactone has been shown to alleviate vascular stiffening and decreases blood pressure in a cohort of 566 patients with uncontrolled hypertension (Liu *et al.* 2018).

In vivo animal studies conducted by our group (Jia *et al.* 2015, 2016) and others (Lyngso *et al.* 2022, Wolter & Jaffe 2023) further demonstrated that ECMR activation prompts oxidative stress and inflammation, ultimately resulting in abnormally increased arterial stiffness. Furthermore, EnNaC signaling participates in activated ECMR-induced vascular stiffening by inhibiting eNOS activation and related NO production (Jia *et al.* 2016). Our recent studies, in an obese mouse model, also indicate that inactivation of AMP-activated protein kinase α and reduced sirtuin 1 are also involved in activation of αEnNaC-mediated EC and arterial stiffening (Sowers *et al.* 2020). Ultimately, these pathophysiological changes appear to promote inflammation and oxidative stress, leading to EC and arterial dysfunction. Consistent with this notion, genetic depletion of either the ECMR or αEnNaC in mice decreased both aldosterone- and Western diet (high fat and refined carbohydrate)- induced increases in endothelial stiffness and impaired eNOS signaling/responses (Jia *et al.* 2018*b*, Sowers *et al.* 2020).

Clinically, pharmacological interventions with MR antagonists have shown some effect in decreasing metabolically-related increases in arterial stiffening and hypertension. The Joint National Committee 8 (JNC8) and the European Society of Hypertension (ESH) recommend that MR antagonists may be considered as the ‘fourth-line’ drug in the treatment of the resistant hypertension (Glicklich & Frishman 2015). Indeed, MR antagonisms with spironolactone or eplerenone effectively reduce blood pressure in patients with therapy-resistant hypertension (Glicklich & Frishman 2015). Meanwhile, amiloride, a blocker of ENaC, has been widely used in human subjects with clinically diagnosed hypertension. Importantly, amiloride also improves endothelial function and reduces vascular stiffness in obese rodents supporting commonality in human subjects and animal models (Martinez-Lemus *et al.* 2017).

### Vascular ECMR/EnNaC in atherosclerosis

Enhanced ECMR and EnNaC activation increase endothelial dysfunction that is the first step in the development of atherosclerosis. The Multi-Ethnic Study of Atherosclerosis, in 6814 community-dwelling adults aged 45 to 84 years, investigated the longitudinal association between serum aldosterone concentrations and coronary artery calcium (Inoue *et al.* 2020). This study demonstrated that aldosterone levels were associated with the increased risk of subclinical coronary atherosclerosis and mortality (Inoue *et al.* 2020). Similarly, patients with primary aldosteronism show greater carotid atherosclerosis than those of hypertensive patients without elevated aldosterone (Liang *et al.* 2022). Moreover, hypertensive subjects with primary aldosteronism have higher rates of CVD events, including acute myocardial infarction and stroke, than those of patients with essential hypertension (Milliez *et al.* 2005).

Several preclinical animal studies have further demonstrated that aldosterone is associated with the development of atherosclerosis. For instance, diet-induced obesity induces endothelial dysfunction with an aldosterone-dependent mechanism, and this effect is reversed by MR antagonist treatment, implicating a role for the ECMR (Marzolla *et al.* 2017). Aldosterone infusion induces inflamed and lipid-rich plaque formation and promotes the development of aortic atherosclerotic lesions in ApoE knockout mice fed an atherogenic diet (McGraw *et al.* 2013). Moreover, aldosterone activates ECMR and induces expression of ICAM-1, promoting leukocyte adhesion to in vitro human coronary ECs (Caprio *et al.* 2008). Interestingly, ECMR contributes to vascular inflammation and atherosclerosis in a sex-specific manner (Moss *et al.* 2019). Recently, one study also indicated that EnNaC participates in high-fat-diet-induced vascular dysfunction and formation of atherosclerosis lesions (Niu *et al.* 2021). Benzamil, a potent ENaC antagonism, attenuates high-fat-diet-induced increases in matrix metalloproteinase 2 and 9, impairment of endothelium-dependent relaxation and formation of atherosclerosis lesions in low-density lipoprotein receptor-deficient mice (Niu *et al.* 2021). Thus, vascular inflammation is an important contributor in activated ECMR/EnNaC signaling-induced EC dysfunction and experimental atherosclerosis.

### Vascular ECMR/EnNaC in CAD and heart failure

Enhanced activation and expression of ECMR/EnNaC impair coronary microvascular dysfunction, decrease coronary flow reserve, and promote development of CAD, cardiac diastolic dysfunction, and heart failure (Joffe *et al.* 2007, Garg *et al.* 2015). Except of coronary artery atherosclerosis, impaired coronary flow reserve is also associated with cardiac damage and is regarded as a powerful independent predictor of cardiac mortality in patients with metabolic syndrome (Garg *et al.* 2015). A randomized, double-blind study of 64 subjects with diabetes but without cardiac ischemia, demonstrated that spironolactone (25 mg daily) significantly improved coronary flow reserve, independent of changes in blood pressure (Garg *et al.* 2015). Eplerenone also improved coronary circulatory function in patients with diabetes and albuminuria who were already receiving angiotensin converting enzyme inhibitor therapy (Joffe *et al.* 2007). Clinical data further found that that plasma aldosterone levels are independent predictors of CAD and that primary hyperaldosteronism patients have a 6-fold increased risk of myocardial infarction even after controlling for blood pressure (DuPont & Jaffe 2017). Large randomized clinical trials including RALES, EPHESUS, and EMPHASIS have shown that MR antagonists reduce morbidity and mortality in both mild and moderately severe heart failure (Jia *et al.* 2015). A randomized, controlled clinical study investigated the efficacy of MR antagonists in patients with cardiac diastolic dysfunction or heart failure with preserved ejection fraction and found that MR antagonists decreased cardiac fibrosis and improved cardiac diastolic dysfunction, suggesting MR signaling as a key contributor of cardiac diastolic dysfunction and heart failure (Pandey *et al.* 2015).

Our recent preclinical study also indicates that cell-specific ECMR signaling mediates Western diet-induced activation of cardiac pro-fibrotic and inflammatory responses that led to cardiac diastolic dysfunction in this diet-induced obese mouse model (Jia *et al.* 2015). To this point, ECMR activation increases EnNaC expression and activation that induce coronary arterial and cardiac stiffness and impaired cardiac relaxation (Sowers *et al.* 2020). Conversely, αEnNaC subunit deletion resulted in significant improvement in in the tissue Doppler *e*′/*a*′ ratio as well as increases in left ventricular filling pressure (*E*/*e*′) and diastolic stiffness (Sowers *et al.* 2020). Related to this, activated EnNaC in metabolic syndrome increases coronary artery endothelial permeability, cardiac oxidative stress, and inflammation (Jia *et al.* 2018*a*). Inhibition of EnNaC with amiloride, in obese female mice, has been shown to decrease cardiac inflammation and fibrosis and improve cardiac diastolic dysfunction (Jia *et al.* 2018*a*).

## Molecular mechanisms of activated ECMR and EnNaC contributing to metabolic syndrome and CVD

### Oxidative stress

Activation of MRs in ECs can increase ROS production and oxidative stress by impairment of mitochondrial and endoplasmic reticulum function and induce vascular dysfunction, atherosclerosis, hypertension, and metabolic syndrome. On the one hand, excessively produced ROS react with cellular components, such as lipids, proteins, and DNA, leading to oxidative damage and cellular dysfunction. On the other hand, increased oxidative stress inhibits activity of antioxidant enzymes, which play a crucial role in neutralizing ROS and preventing oxidative damage. ECMR signaling activates NADPH oxidase, which is one of the important enzymes in the generation of ROS production and excessive oxidative stress. To this point, aldosterone increases expression of NADPH components Nox2, p22phox, and p47phox through an ECMR-dependent mechanism (Jia *et al.* 2015, 2016). Meanwhile, both Nox1 and Nox4 are also associated with ROS-sensitive aldosterone production (Jia *et al.* 2015, 2016). Moreover, excessive ROS activates redox-sensitive serine kinases to activate serine phosphorylation of IRS-1 and impair insulin metabolic signaling (Jia *et al.* 2015, 2016). Our previous studies have also demonstrated that ECMR gene deletion prevents Western diet-induced increases in 3-nitrotyrosine production in vascular and cardiac tissues, which is a marker for peroxynitrite formation, the lipid peroxidation chain reaction, and excessive tissue oxidative stress (Jia *et al.* 2015, 2016).

While oxidative stress increases ENaC expression and activity and contributes to salt-sensitive hypertension, increased EnaC also leads to increased ROS production. For instance, EnaC promotes oxidative stress in dendritic cells in salt-induced hypertension (Barbaro *et al.* 2017). In this regard, Na^+^ enters cells and initiates calcium influx through α/γ-EnaC and the sodium hydrogen exchanger 1, leading to activation of protein kinase C, phosphorylation of p47*^phox^*, and association of p47*^pho^*with gp91*^phox^* (Barbaro *et al.* 2017). The assembled NADPH oxidase produces superoxide with subsequent formation of immunogenic isolevuglandin (IsoLG)-protein adducts and hypertension (Barbaro *et al.* 2017). Interestingly, EC-specific αEnNaC also mediates aldosterone- and Western diet-induced increases in 3-nitrotyrosine, Nox2, and oxidative stress and associated arterial and cardiac dysfunction (Jia *et al.* 2018*a*,* b*).

### Inflammation

Enhanced ECMR activity induces vascular leukocyte adhesion, infiltration, and related proinflammatory tissue responses. To this point, activated ECMRs increase EC-derived inflammatory adhesion molecules, including selectins, VCAM-1, and ICAM-1, which promote leukocyte–endothelial adhesion and facilitate transendothelial infiltration into the vasculature and other neighbor tissues. In vitro human coronary ECs, aldosterone increases transcription and protein expression of ICAM1 that promote leukocyte adhesion to ECs in an MR-dependent manner (Caprio *et al.* 2008). In vivo, aldosterone promotes vascular ICAM1 mRNA expression and associated atherosclerosis that is prevented in ICAM1-deficient ApoE-KO mice (Marzolla *et al.* 2017). Activated ECMR also increases expression of proinflammatory adipokines, including tumor necrosis factor-α (TNFα), interleukins (ILs), and monocyte chemoattractant protein-1 (MCP-1) to directly impair insulin metabolic signaling and related metabolic syndrome. Enhanced ECMR signaling also induces activation of the T helper (Th) cell inflammation response and macrophage M1 polarization (Jia *et al.* 2021). However, MR antagonism has been found to inhibit the expression of TNFα, MCP-1, and IL-6, as well as the macrophage M1 marker markers CD68 and CD11c (Jia *et al.* 2021). Our recent data further demonstrated that gene deletion of ECMR inhibits pro-inflammatory cytokines, such as IL17 and CD11b while showing increased M2 marker expression (CD206 and IL-10) in aortic and cardiac tissues in diet-induced obesity (Jia *et al.* 2015, 2016).

EnNaC induces oxidative stress to increase the inflammatory response (Barbaro *et al.* 2017). EnNaC also increase vascular stiffness that promotes EC cell expression of adhesion molecules including ICAM-1 and VCAM-1, which, in turn, induces leukocyte/macrophage migration, adhesion, and infiltration (Jia *et al.* 2018*b*). Indeed, our previous study demonstrated that Western diet decreased arterial expression of tight junction proteins (claudin 5 and occluding), resulting in macrophage recruitment and cardiac fibrosis (Jia *et al.* 2018*a*). Conversely, inhibition of EnNaC with amiloride or αEnNaC gene deletion attenuated diet or aldosterone-increases in endothelium permeability and increased expression of CD68, IL-1, and IL-6, as well as decreasing infiltration of monocytes/macrophages and migration within vascular and cardiac tissue (Jia *et al.* 2018*a*,* b*).

### Lipid disorders

Enhanced MR activation in overnutrition or obese individuals promotes excessive free fatty acid (FFA) uptake, lipid metabolic disorders, and insulin resistance. A clinical study indicated that there was a strong negative correlation between plasma aldosterone and high-density lipoprotein cholesterol levels in 30 patients with metabolic syndrome (Goodfriend *et al.* 1995). Conversely, 6 weeks of eplerenone treatment decreased plasma triglyceride levels in patients with diabetes (Joffe *et al.* 2007). Our (Habibi *et al.* 2022, Hulse *et al.* 2022) and other (Ramirez *et al.* 2013) preclinical studies further demonstrated MR antagonisms prevented Western diet- or palmitic acid-induced increases in FFA uptake, ectopic lipid droplet accumulation, and lipid metabolic disorders in liver, skeletal muscle, and heart. Moreover, CD36 is involved in the activated MR-induced excessive FFA uptake and ectopic lipid accumulation associated with systemic and tissue-specific insulin resistance (Hulse *et al.* 2022). Related to this, CD36 is a fatty acid translocase that enhances cellular FFA uptake and corresponding development and progression of lipid disorders, atherosclerosis, and metabolic syndrome (Hulse *et al.* 2022). Importantly, CD36 is present in exosomes, which shuttle CD36 between neighboring cells, and has been implicated as a mediator in diet-induced lipid metabolic disorders and metabolic syndrome (Garcia *et al.* 2019, Yan *et al.* 2021). Therefore, it is possible the ECMR signaling impacts CD36, EC-derived exosomes, and associated lipid metabolic disorders. To date, few studies have, however, investigated the role of EnNaC in lipid metabolism. One study has shown that endogenously expressed ENaC is present in lipid rafts in A6 cells (Hill *et al.* 2002). These cholesterol-rich domains within the plasma membrane serve metabolic and signaling processes in lipid physiology and pathophysiology. Thus, it will be important to clarify the pathophysiological relationships between ECMR/EnNaC activation and lipid disorders.

### Sexually dimorphic roles of ECMR/EnNaC in the metabolic syndrome and CVD

Compared to premenopausal women, postmenopausal women appear more susceptible to the deleterious effects of CVD. This is thought to be, in part, due to decreased plasma estrogen levels (Faulkner & Belin de Chantemele 2018, Wolter & Jaffe 2023). In overweight females, plasma aldosterone levels are elevated, and increased aldosterone is linked to cardiac hypertrophy and remodeling in females but not males (Vasan *et al.* 2004). As mentioned earlier, our recent preclinical data also demonstrated that 16 weeks of Western diet feeding significantly increased plasma aldosterone levels to 3166 pmol/L (Aroor *et al.* 2019), which would be expected to further activate MRs in vascular, skeletal muscle and liver tissue with resultant increases in systemic and tissue-specific insulin resistance, as well as metabolic syndrome (Jia *et al.* 2021). Further, a preclinical, high-fat-diet-induced obesity, mouse model study showed that while serum estradiol levels were significantly decreased in hyperlipidemic females, serum aldosterone and corticosterone levels were significantly increased as compared to similarly fed males (Davel *et al.* 2018). Meanwhile, leptin induces hypertension and endothelial dysfunction via aldosterone-dependent mechanisms in female mice, suggesting that obesity leads to CVD via sex-specific mechanisms (Huby *et al.* 2015). Interestingly, 17 β-estradiol inhibits aldosterone synthesis by anestrogen receptor β mediated mechanism (Caroccia *et al.* 2014). Moreover, there was a sex difference in the mechanism of high-fat-diet-induced mesenteric vascular endothelial dysfunction (Davel *et al.* 2018). In this regard, obesity or hyperlipidemia decreased NO-dependent vasodilation in males, whereas endothelium-derived hyperpolarization was significantly impaired in females (Davel *et al.* 2018). NO-induced dilation was impaired by obesity and hyperlipidemia in males with no apparent role of ECMR (Davel *et al.* 2018). In contrast, endothelial dysfunction induced by obesity and hyperlipidemia in females was due to a decline in endothelium-dependent hyperpolarizing factor-mediated mechanisms and the improved dilation in female ECMR knockout mice was mediated by a compensatory increase in NO (Davel *et al.* 2018). Chronic aldosterone infusion or Western diet induced endothelial dysfunction, aortic stiffening, and cardiac dysfunction in female mice that were prevented in ECMR or αEnNaC knockout mice (Jia *et al.* 2016, 2018b). Moreover, gene deletion of ECMR did not impact plaque size but decreased aortic plaque inflammation by 50%, specifically in males (Moss *et al.* 2019). Female mice have larger plaques with less plaque inflammation than males, but these parameters were unaffected by ECMR knockout in females (Moss *et al.* 2019). Indeed, clinical studies in humans also support the sexual dimorphic role of MR in overnutrition/obesity-induced endothelial dysfunction. In this regard, a prospective cohort study of African American patients with documented hypertension found that women taking spironolactone had improved blood pressure control, which was not seen in spironolactone-treated men (Clemmer *et al.* 2019). Interestingly, estrogen signaling through estrogen receptor α increases EnNaC activity to a larger extent in females compared with males, and increased EnNaC activity is associated with reduced nitric oxide and EC stiffening (Padilla *et al.* 2019). As sexual dimorphisms in ECMR/EnNaC function have been implicated in EC dysfunction and metabolic syndrome, it will be necessary to thoroughly investigate the interaction between sex and the contribution and potential therapeutic potential of ECMR/EnNaC in patients with metabolic syndrome.

### Targeting ECMR/EnNaC in metabolic syndrome and CVD

MR and ENaC antagonists, including eplerenone and spironolactone, amiloride and triamterene, are currently approved for clinical hypertensive and heart failure patients. MR antagonists have also been shown to improve glucose metabolism and prevent insulin resistance and metabolic syndrome. For instance, adrenalectomy or spironolactone treatment improves insulin resistance in patients with primary aldosteronism (Catena *et al.* 2006). One preclinical study also demonstrated that administration of FAD286, an aldosterone synthase inhibitor, reduced plasma aldosterone levels and improved metabolic parameters in Zucker diabetic rats (Hofmann *et al.* 2016). Our recent studies also indicated that inhibition MRs with spironolactone improves insulin-stimulated glucose transport, systemic and tissue insulin sensitivity (Habibi *et al.* 2022, Hulse *et al.* 2022). However, while the impact of MR antagonists on patients with metabolic syndrome is under investigation, no definitive data are available. Recent clinical studies evaluating the nonsteroidal MR antagonists, including esaxerenone, apararenone, and finerenone, in patients with hypertension and heart failure have provided encouraging results. For instance, finerenone can activate brown adipose tissue and thus prevented overnutrition- or obesity-induced insulin resistance and metabolic syndrome (Marzolla *et al.* 2020). Clinical studies further suggest that finerenone has higher affinity for the MR than spironolactone and lacks affinity for other steroid receptors, making it a potential new drug in metabolic syndrome (Pandey *et al.* 2022). Although our (Martinez-Lemus *et al.* 2017, Jia *et al.* 2018*a*) and other (Nizar *et al.* 2016) preclinical studies indicate that ENaC inhibitors have a beneficial role in improving glucose metabolism and insulin sensitivity, and preventing metabolic syndrome, clinical data are lacking to provide evidence for the efficacy of ENaC inhibitors in subjects with metabolic syndrome.

## Conclusion

Enhanced endothelial ECMR and EnNaC signaling appear to be important contributors in the development of metabolic syndrome, which includes a constellation of factors such as insulin resistance, dyslipidemia, obesity, and associated CVD. Metabolic syndrome related CVD incudes arterial stiffening, hypertension, atherosclerosis, CAD, and heart failure. Heightened oxidative stress, inflammation, and lipid metabolic disorders participate in these pathophysiological processes. Excessive aldosterone/ECMR/EnNaC signaling in the pathogenesis of metabolic syndrome may be especially important in women since premenopausal females with obesity and diabetes lose hormonal metabolic protection and have greater propensity to develop metabolic syndrome. However, further basic and clinical research is needed to fully understand the role of the endothelial ECMRs/EnNaC in metabolic syndrome development and therapeutic strategies.

## Declaration of interest

The authors declare no conflict of interest.

## Funding

This research was supported by the National Institute of Diabetes and Digestive and Kidney Diseases (R01 DK124329) and an American Diabetes Association Innovative Basic Science Award (1-17-IBS-201) to G.J. Dr Sowers received funding from NIH (R01 HL73101-01A and R01 HL107910-01). Dr Hill receives funding from NIH (RO1HL085119).
